# Monthly Summary

**Published:** 1894-01

**Authors:** 


### Monthly Summary.

NUMBER OF DENTAL COLLEGES AND STUDENTS.—Dr. Ottofy reports to the American Dental Association that there have been established since last year the Dental Department of Tennessee Medical College, at Knoxville, Tenn.; the Chicago Tooth Saving Dental College, at Chicago, Ill.; the Dental Department of the Homœopathic College, at Cleveland, Ohio; the Dental Department of the Western Reserve University, at Cleveland, Ohio; and the Dental Department of the University of Buffalo, making an increase of five over the previous year. The total number now in operation is thirty-eight. The number of graduates in 1891 was 1,241; in 1892 there were 1,483. The average in the last seven years is 904 graduates per annum. The superfluity of many of these colleges was well demonstrated last year, when attention was called to the fact that it required the strength of fifteen colleges, more than one-third of the number, to graduate less than 100 students, eight more turned out 150, while twelve colleges out of thirty-three graduated more than three-fourths of the entire number. We have here a tabulated statement of all the dental colleges. Two of the new colleges have held no commencement. It required this year the force of fourteen full fledged colleges, with their equipments, faculties, etc., supposed to be perfect, to graduate ninety-one students, an average of six and one-half each. In fact, eleven colleges together graduated fifty-one students. Who can estimate the terrible loss to the profession, were eleven colleges to be suddenly taken off the list? Eight hundred and fifty-one were passed from ten institutions, or a little more than one-quarter of the entire number of colleges graduating almost two-thirds of the list. The three years' college course does not seem to have had the ill effect so strenuously predicted. On the contrary, the number of students entering the colleges, though smaller, is superior. They fully appreciate the additional advantages gained. The number of years which a student should devote to dentistry will soon be placed at four years of nine months each Any young man, with a proper preliminary education, will find that time sufficient to become proficient. There are now about one hundred and thirty local societies in the United States, with an aggregate membership of nearly five thousand.—*International.*

---

FOR THE TEETH—SOME EXCELLENT RULES TO FOLLOW IN THE CARE OF THEM.—One of the most skillful dentists in New York gives these rules for the care of the teeth:

Use a soft brush and water the temperature of the mouth. Brush the teeth up and down in the morning, before going to bed, and aftet eating, whether it is three or six times daay. Use a good tooth powder twice a week, not oftener, except in case of sickness, when the acids from a disordered stomach are apt to have an unwholesome effect upon the dentine. Avoid all tooth pastes and dentifrices that foam in the mouth; the latter is a sure sign of soap, and soap injures the gums, without in any way cleansing the teeth.

The very best powder is cf precipitated chalk; it is absolutely harmless and will clean the enamel without affecting the gums. Orris root or a little wintergreen added gives a pleasant flavor, but in no way improves the chalk. At least a quart of tepid water should be used in rinsing the mouth. A teaspoonful of Listerine in half a glass of water used as a wash and gargle after meals is excellent; it is good for sore or loose gums; it sweetens the mouth, and is a valuable antiseptic, destroying promptly all odors emanating from diseased gums and teeth. Coarse, hard brushes and soapy dentifrices cause the gums to recede, leaving the dentine exposed. Use a quill pick if necessary after eating, but a piece of waxed foss is better. These rules are worth heeding.

Be assured of the genuine Listerine by purchasing an original bottle.

---

DEATHS UNDER ANÆSTHETICS.—Gurlt reported to the last Surgical Congrese at Berlin, the following statistics of deaths under anæsthetics. They are made up from observations of sixty-two operators, who anæsthetized 109,196 persons with thirty-nine fatal results, showing one death to 2,800 narcoses. The following were the anæsthetics used.

Chloroform, 94,123 narcoses; 36 deaths.

Ether, 9,431 narcoses; no deaths.

Ether and chloroform, 2,881 narcoses; 1 death.

Ether and alcohol, 1,381 narcoses; no deaths.

Bromoform with ethyl bromide, 2,151 narcoses; 1 death.

Pental, 210 narcoses; 1 death.

In 2,913 cases the narcosis lasted over an hour, in an operation for utero-vaginal fistula, 4½ hours; in a case of tetanus, 9 hours. In 25 cases, of which post-mortem examinations were made, cardiac diseases were found. The author urges careful examination of the heart before administering chloroform.—*Cond. Extracts.*

---

THE ADMINISTRATION OF ANESTHETICS —The following deductions ("Lancet," No. 3642, p. 1498) have been arrived at by the Commission appointed by the "Lancet" to investigate from a clinical standpoint the subject of the administration of chloroform and other anesthetics: The death-rate under anesthetics has heretofore been unduly high, and may be lowered by improved methods and greater care. Ether is the safest anesthetic in temperate climes for general surgery, when properly given from an inhaler permitting graduation of the strength of the vapor. Nitrous oxide gas should be employed for minor surgery. Chloroform is a comparatively safe body when given by a carefully trained person, but is not in any case wholly devoid of risk. No age or nation is free from danger under anesthetics. The perils of anesthesia, however slight, demand that the undivided attention of a duly qualified and trained medical man should be given to the administration of the anesthetic.—*Med. and Surgical Reporter.*

---

THE USE OF COCAINE.—1. Amount of cocaine used must be in proportion to extent of surface it is desired to anæsthetize. In no case should the quantity exceed one grain and three-quarters.

2. Cocaine should never be used in cases of heart disease, pulmonary disease, or in persons of highly nervous temperament.

3. In injecting cocaine, the intradermic method is preferable to hypodermic, By injecting into, not under mucous membrane or skin, the risk of entering a blood vessel is avoided.

4. During injection the patient should always be in a recumbent position; in operations upon the nose and throat, the head should not be raised until anæsthesia is complete.

5. It is of great importance that cocaine should be pure, since its combinations with certain other alkalies result in poisonous compounds.—*Brooklyn Med. Journ.*

---

HYGIENE OF HAIR DRESSING AND BARBER SHOPS.—By Dr. Angel Contreros, Pueblo, Mexico.—The disease which persons are most liable to contract in barber shops is scurf, and the author therefore touched on what appeared to him the most important points in the consideration of this matter. Scurf is understood to be a disease of the capillary system caused by the presence of vegetable parasites. The disease can be transmitted by some animals which already have it; but the contagion most commonly takes place from person to person; and in families, in educational establishments, and in barracks the disease assumes an endemic character. Several times the center of this propagation has been found in a hair dresser's or a barber's shop and has arisen from the use of instruments which have been badly cleansed. Barbers and hair-dressers ought therefore to be very careful in cleansing the utensils which have served for one person before they are employed on another. All the utensils should be subject to the action of heat for a space of ten minutes in a vessel or receptacle at a temperature of 120 degrees, and the razors in an oil bath.—*Med. and Surg. Reporter.*

---

IODIDE OF POTASH IN HEADACHE.—In the "Anales of Del Circulo Medico Argentino," Tomo XVI, No. 5, 1893, the iodide of potash, in small and frequently repeated doses is recommended as an efficacious remedy in headache. Two grains in an ounce of water every quarter hour will relieve completely, in a short space of time, frontal headache accompanied by sensations of heat and chilliness, languor, general malaise, repugnance of food and nausea. These headaches are from definite or special causes and may be regarded as sympathetic headache with a slight syphilitic element, which is either hereditary or acquired. The iodide of potash administered in this manner, often exercises a marvelous action.

---

PINUS CANADENSIS cannot be too highly recommended as an application to burns, especially when very extensive, the skin being entirely removed. A week solution in glycerin is squeezed from a sponge over the denuded surface, which is then dressed with some soft ointment, either with or without the pinus canadensis. Pain immediately abates, and the healing process is wonderfully rapid. The solution must be freshly applied, as often as the dressings are renewed.—*Southern Journal.*

